# Oyster Biodeposition Alleviates Sediment Nutrient Overload: A Case Study at Shenzhen Bay, China

**DOI:** 10.3389/fmicb.2021.716201

**Published:** 2021-11-10

**Authors:** Autif Hussain Mangi, Qi Yan, Xiao Song, Junting Song, Xia Lan, Jin Zhou, Zhong-Hua Cai

**Affiliations:** ^1^Shenzhen Public Platform for Screening and Application of Marine Microbial Resources, Institute for Ocean Engineering, Shenzhen International Graduate School, Tsinghua University, Shenzhen, China; ^2^School of Life Sciences, Tsinghua University, Beijing, China

**Keywords:** oyster biodeposition, sediment nutrient, alleviative effect, eutrophication, Shenzhen Bay

## Abstract

Oysters are ecological engineers, and previous studies have examined their role as competent facilitators of ecological restoration. However, the decisive role of oysters in the aquatic environment is still debatable because oyster biodeposition (OBD) may also increase the nutrients enriched in sediments. In order to better interpret this problem, we sampled sediment cores from representative oyster culture areas and uncultured areas in Shenzhen Bay. The results have shown that the TOC (total organic carbon) and TN (total nitrogen) decreased significantly (*p* < 0.05) at the surface sediment layer (0–20-cm deep) and the sediment layer (20–40-cm deep) of the oyster site compared with the reference site. The decreased TOC and TN were also observed at 60- to 100-cm sediment depth in the oyster site. This indicated that the OBD significantly impacted the concentration of TOC and TN in the sediment. To confirm the alleviative role of OBD, we conducted stable isotope (δ^13^C and δ^15^N) analyses, which further demonstrated the presence of heavier and less lighter forms of organic carbon and nitrogen sediment. The surface sediment layer (0–20 cm) at the oyster site showed 8% more δ^13^C‰ compared with the control site (*p* < 0.05), reflecting the reduction in the TOC. In order to reveal the potential microbial mechanisms involved in OBD, we performed a functional analysis using the Geochip5 advanced microarray technology. Regarding carbon metabolism, we observed that genes (encoding pullulanase, glucoamylase, exoglucanase, cellobiase, and xylanase) involved in the degradation of relatively labile C-based molecules (e.g., starch, cellulose, and hemicellulose) were highly represented in an experimental area (*p* < 0.05). In addition, microbes in the experimental area exhibited a greater capacity for degrading recalcitrant C (e.g., lignin), which involves glyoxal oxidase (*glx*), manganese peroxidase (*mnp*), and phenol oxidase. Among the genes controlling nitrogen metabolism, the genes involved in denitrification, assimilation, ammonification, and nitrification were differentially expressed compared with the control area. These results indicated that microbial metabolic roles might have enhanced the C/N-flux speed and reduced the overall nutrient status. We concluded that OBD alleviates sediment nutrient overload under oyster farming from a microbial ecological perspective in a rapidly urbanized coastal area.

## Graphical Abstract

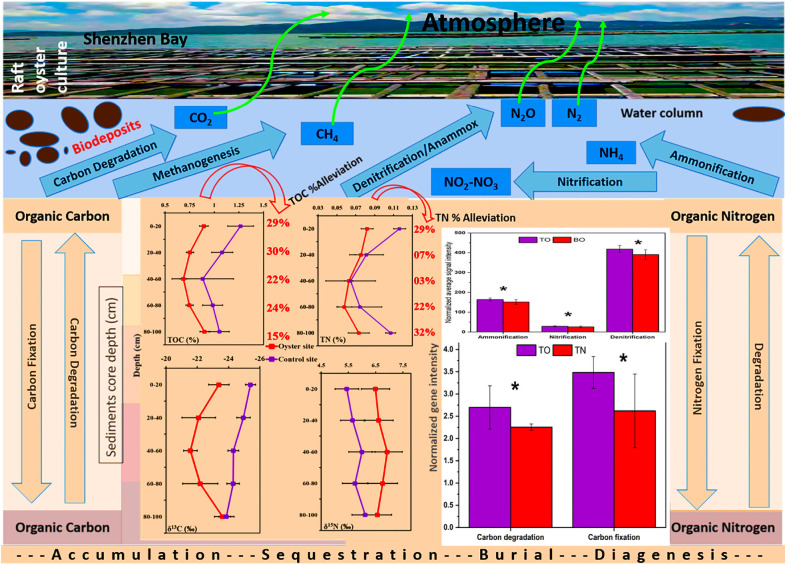

## Introduction

Eutrophication and environmental pollution are severe threats to coastal ecosystems worldwide (Lotze et al., 2006). Anthropogenic activity significantly influences organic matter (OM) distribution and deposition in coastal areas, especially in bays (Gao et al., 2012). The main reason for this is because various nutritive elements such as C, N, and P can accumulate to undesirable levels, and when carbon and nitrogen enter the marine system by various sources, they can accumulate to such high local concentrations that eutrophication may result (Nixon, 1995; Bowen and Valiela, 2001; Kemp et al., 2005). Many adverse consequences result from eutrophication, such as harmful algal blooms (Paerl, 1997; Glibert et al., 2005), increased hypoxic events (Diaz and Rosenberg, 1995; Rabalais et al., 2010), and loss of benthic habitats (Diaz and Rosenberg, 1995, 2008; Jennifer et al., 2003).

Filter feeders have the ability to reduce eutrophication. When oysters and other bivalves feed, they remove particulate organic matter and subsequently decrease the turbidity of water (Cunha et al., 2019). Shellfish, particularly oysters, are referred to as ecosystem engineers because they supply many ecosystem services such as increased biodiversity, healthy fisheries, and water purification (Newell, 2004; Grabowski and Peterson, 2007; Loren et al., 2007; Peterson et al., 2007; Marzocchi et al., 2021). In contrast, ingested but undigested phytoplankton and particles can deposit on the sediment superficies as nutrient-enriched pseudofeces (Newell, 1996).

The effects of nutrient filtration by these filter feeders have been controversial for a long time. The main reason is that the excreta of filter feeders are concentrated in sediments. Sediments act as a sink of nutrients but also may serve as a source of nutrients in the water, leading to eutrophication (Gautreau et al., 2020). Hence, it is necessary to evaluate the influence of mariculture on the preservation status of organic matter and the primary source–flux response in sediments.

Carbon and nitrogen contained in oyster biodeposits may have different biogeochemical fates, depending upon their environmental situation. Documenting environmental drivers of carbon and nitrogen dynamics in sediments is required to foresee the effect of oyster biodeposition (OBD) on eutrophication. Organisms that feed on deposits can consume fresh organic carbon and nitrogen incorporated in biodeposits, and then disturb the sediments for a brief or an extended period, or decompose to dissolved organic carbon and nitrogen followed by mineralization (Newell et al., 2005; Giles and Pilditch, 2006; Forrest et al., 2009).

Microorganisms are the most direct driving force that affects the amount of matter in sediment. Sedimentary microorganisms can decompose and mineralize nutrients and the organic matter in the biodeposition process (Newell and Koch, 2004), and can also be taken up by various microbes (Cornwell et al., 1999). The previous viewpoint deemed that biogenic elements could be released from surface sediments into the overlying water column in different inorganic or organic forms and increase the eutrophication pressure and water quality deterioration. However, it remains unclear whether biodeposits boost or undermine nutrient enrichment in the coastal environment because previous research lacks information regarding the determination of OBD influence on carbon–nitrogen dynamics and their associated biological cycling mechanism during a vertical analysis of sediments in urbanized areas. Therefore, it is essential to clarify how OBD influences sediment carbon and nitrogen dynamics and to determine the microbial metabolic roles involved in the carbon and nitrogen cycling process.

In the current study, we tested the hypothesis that the sediment carbon and nitrogen concentration are significantly altered under the influence of the OBD status. We suggest that the oyster farming at Shenzhen Bay might accelerate the metabolism of nutrients and other microbial activities. Our purpose was to confirm the positive role of OBD and to determine the related microbial mechanisms involved in the regulatory processes of the carbon and nitrogen cycle from the microbial perspective. We chose Shenzhen Bay as the study area because it is an ideal place to determine the status of anthropogenic influence and eutrophic events during the pre- and post-urbanization period using its sediments as an indicating biomarker. In addition, there is more than a 13-km-long oyster culture area, which supplied us with excellent experimental conditions *in vivo*.

As for the experimental tools, GeoChip 5.0 advanced microarray technology and continuous flow–isotopic ratio mass spectrometer (CF-IRMS) techniques were simultaneously employed to address two key questions: (1) What is the impact of OBD on nutrients (especially the C/N-flux) in the sediments of the oyster farming area, and (2) How do microbes participate in the alleviation of the nutrient overload state? The link between microbial functional genes and oyster biodeposits behind nutrient dynamics was also evaluated. This study may have potential implications on the ecological health of a bay in terms of eutrophication, C burial, and preservation.

## Materials and Methods

### Study Area and Sediment Sampling

**FIGURE 1:**
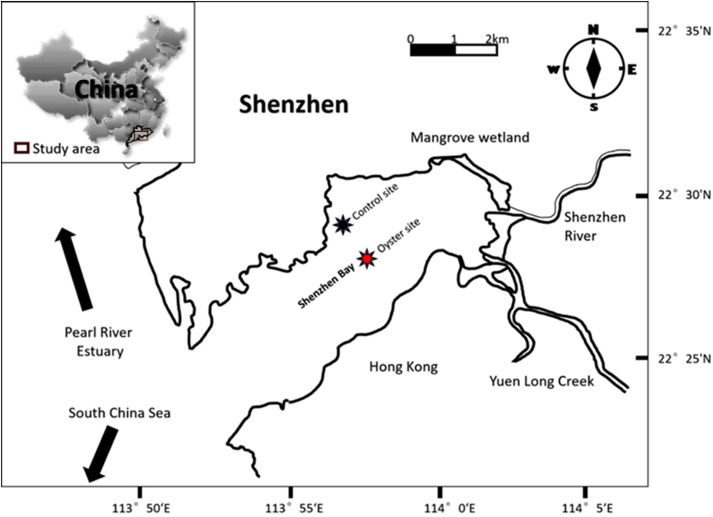
Map of Shenzhen Bay. The red star shows the oyster farming area (site under oyster biodeposition (OBD) influence), and the dark purple star indicates the control site (area without any shellfish culture).

The study site lies in Shenzhen Bay (22° 27′ 55.188″ N, 113° 57′ 56.2932″ E), which is located on the east shore of the Pearl River Estuary, Shenzhen, Guangdong Province, China (Figure 1). Shenzhen Bay is a typically shallow, semi-enclosed bay with an average water depth of 4 m and a tidal height of approximately 1.4 m. Oyster culture has been in operation in Shenzhen Bay for the last three decades. There are approximately 10,000 floating oyster rafts that occupy a 13-km^2^ area of Shenzhen Bay. The main species of oyster is *Crassostrea hongkongensis*. According to research on the chronology of Shenzhen Bay, the average sedimentation rate ranges between 0.92 and 1.10 cm/a, and therefore, the 50-cm-long sediment core was dated back to the year 1958. The clay ranges from 54.4 to 70.0% with a mean of 66.3%, silt from 29.9 to 40.4% with a mean of 32.8%, and sand from 0.0 to 5.8% with a mean of 0.9%.

Sediment samples were collected from two distinct Shenzhen Bay sites: the oyster farming area (test site) and the non-farming area (control or reference site) during a cruise in November 2018. At each location, eight manual cores were taken by a 100-cm-long polyvinyl sediment collector, and each sampled core was rapidly divided into five distinct layers of equal measure (20 cm) from the top to the bottom end. The sediments were sealed in airtight plastic bags and maintained at 4°C until they could be immediately brought back to the laboratory for further analysis.

Each sediment sample was subdivided into independent portions for GeoChip analysis, stable isotope detection, and nutrient determination. Suspended particulate matter was collected by filtering 500–1,000 ml of surface water from the bay with a piece of mesh having a pore size of 200 μm. The temperature, pH, salinity, turbidity, dissolved oxygen, and chlorophyll-*a* concentration were measured with an automated instrument (YSI 6600, Germany).

### Determination of Organic and Inorganic Nutrients in the Sediment

The levels of dissolved inorganic nutrients (NO_3_^–^, NO_2_^–^, and NH_4_^+^) were determined by a discrete chemistry analyzer (CleverChem; Anna, Germany) following a standard colorimetric method (Grasshoff et al., 1985). Full protocol details are provided at http://cmore.soest.hawaii.edu. Standards (purity 99%) were purchased from Shanghai Macklin Biochemical Co., Ltd. (China). The organic carbon and nitrogen were analyzed by drying the sediment in an oven at 60°C overnight, followed by an acid wash to remove an inorganic portion. The analysis was performed using an elemental analyzer (Flash EA-HT 1112 Thermo Scientific, Germany) with analytic precision of < 0.5%.

### δ^13^C and δ^15^N Analysis by Continuous Flow–Isotopic Ratio Mass Spectrometry

The δ^13^C and δ^15^N were analyzed by drying the sediments, followed by an acid wash to exclude inorganic content. Stable isotopic signatures were analyzed by a continuous flow–isotope ratio mass spectrometer (Delta V Advantage, Thermo Fisher Scientific, Inc., Bremen, Germany). The δ^13^C and δ^15^N values of OM in the surface sediments and biodeposited samples were analyzed using an isotope ratio mass spectrometer (DELTAplus XL). Stable isotope ratios were assigned in δ notation expressed by the deviation from a standard reference material in parts per mil (‰): δ^13^C or δ^15^N (‰) = (R_sample_/R_standard__–_ 1) × 1,000, where R denotes ^13^C/^12^C or ^15^N/^14^N. PeeDee Belemnite was used as a reference standard for carbon with an analytical precision of < ± 0.1‰, whereas atmospheric N_2_ was used as a reference standard for nitrogen with an analytical precision of < ± 0.2‰ (Ke et al., 2017).

### Detection of the Labile and Recalcitrant Nature of Organic Matter

Labile and recalcitrant carbon and nitrogen were determined by a reported standard method in which the hydrolysis of organic matter was performed in two sequential steps using strong sulfuric acid (Oades et al., 1970; Rovira and Vallejo, 2000). Briefly, 100 mg of sediment was treated with 20 ml of 2.5 M H_2_SO_4_ for hydrolysis in sealed Pyrex tubes and maintained in an oven at 105°C for 30 min. The hydrolyzate portion was separated by centrifugation, and the remaining residue was washed with purified water. After drying, 2 ml of 13 M H_2_SO_4_ was placed in the tubes, and they were kept in an electric shaker for 18 h. After that, the acid was diluted with purified water to decrease its concentration to 1 M, and the residue was heated in the oven for 3 h at 105°C for further hydrolysis. Finally, the supernatant was recovered by centrifugation, and the residue was dried for analysis of carbon and nitrogen with a Flash EA-HT Thermo Scientific, Germany Elemental analyzer.

The recalcitrance index (RI) can be defined as:

*RIC = (unhydrolyzed C or N/total OC or N) × 100*,
*RIN = (unhydrolyzed C or N/total OC or N) × 100* (Rovira and Vallejo, 2000),

where RI denotes the reference index of carbon/nitrogen, and OC and N denote organic carbon and nitrogen, respectively.

### Extraction and Purification of DNA From the Sediment

Briefly, 0.5 g of each sediment sample was extracted to obtain the microbial community genomic DNA, and the samples were sequentially ground in liquid nitrogen. The fine samples were then further treated with NaC1_2_H_2__5_SO_4_ for cellular breakdown (Hurt et al., 2001). Total DNA was directly extracted from the sediment using a DNA Kit (Power Water, United States) according to the instructions of the manufacturer. The quality of the extracted DNA was estimated by the ratio of OD_260_/OD_280_ to verify that the value was > 1.8 (Nanodrop^TM^ 2000).

### GeoChip-Based DNA Microarray Hybridization, Scanning, and Data Processing

GeoChip 5.0 was used to analyze the functional potential of sediment microbial communities. The purified DNA (500 ng) was labeled with Cy 3 as previously described (He et al., 2012). The labeled DNA was then re-suspended in hybridization solution [42 μl; 1 × HI-RPM hybridization buffer, 1 × Acgh blocking, 0.05 μg/μl of Cot-1 DNA, 10 pM universal standard DNA, and 10% formamide (final concentrations)]. GeoChip hybridization was carried out at 67°C in an Agilent hybridization oven for 24 h. After hybridization, the slides were washed with Agilent Wash Buffers I and II for 5 and 1 min, respectively. The arrays were then scanned with a NimbleGen MS200 Microarray Scanner (Roche NimbleGen, Inc., Madison, WI, United States). The images were extracted by the Agilent Feature Extraction program. Poor-quality spots or those with a signal-to-noise ratio of less than 2.0 were removed. Positive signals were normalized within each sample and across all samples, and then any spots only detected in one sample were removed. The processed data were then used for further analysis (He et al., 2012).

The raw data were uploaded to the microarray analysis pipeline^1^ and analyzed as previously described (Yan et al., 2015). In brief, the following steps were performed: (1) spots with a signal-to-noise ratio less than 2 were removed due to their poor quality; (2) for each sample, intensities greater than 1 were transformed *via* algorithm and divided by the mean signal intensity; and (3) the microbial metabolic potentials were further assessed by the relative signal intensity, which was the standardized signal intensity normalized again based on the number of probes per sample (Chan et al., 2013). A dissimilarity test by permutational multivariate analysis of variance (PERMANOVA) was performed using Bray–Curtis dissimilarity with logarithm-transformed data to compare each dataset or sub-dataset of GeoChip data. Monte Carlo permutation was used to test the significance of the statistics. The analyses were performed using the Vegan package (v.2.2.0) in R software version 3.1.2. Significant differences were indicated at *p* < 0.05 or *p* < 0.01.

### Data Analysis

Quantities are expressed as the mean ± standard deviation (SD). One-way analysis of variance (ANOVA) was carried out to determine the effects on each parameter, followed by Tukey’s tests. To analyze the differences between the oyster site and control site, *t*-tests were used. *p* < 0.05 was considered statistically significant. All statistical analyses were performed using SPSS 11.0 software (IBM SPSS Statistics for Windows, Version 20.0, IBM Corp., Armonk, NY, United States). The graphs were drawn using OriginPro 2021b software (OriginLab Corporation).

## Results

### Total Organic Carbon and Total Nitrogen

**FIGURE 2:**
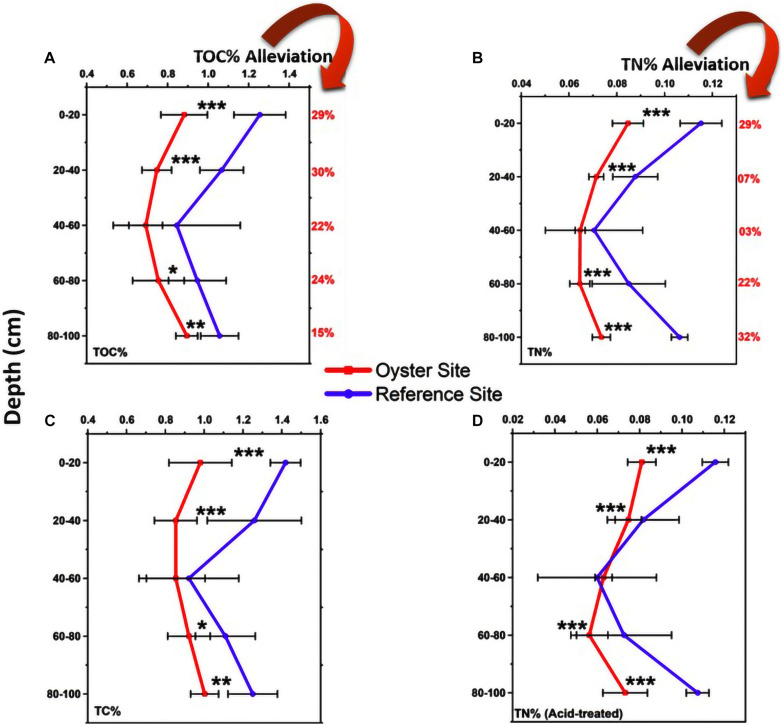
Total carbon and nitrogen. **(A)** TOC%: Total organic carbon is shown in five sediment layers from top to bottom at both the oyster site and control site. The **(B–D)** are total nitrogen (TN)%, TC%, and TN% (acid-treated), respectively. In this image, the red color shows the nutrient status at the oyster site, and the purple color shows the nutrient change in the reference site. Error bars denote standard deviation (SD) of the mean (*n* = 90). The statistical significance were set at three levels: *P* < 0.05 (*), *P* < 0.01 (**), and *P* < 0.001 (***).

The basic physical and chemical parameters (temperature, salinity, pH value, etc.) and nutrient parameters (NH_4_^+^, NO_3_^–^, NO_2_^–^, and PO_4_^3–^) are shown in Supplementary Figures 1, 2. The total organic carbon (TOC) level of the oyster culture area was sediment depth dependent (Figure 2A). The proportion of TOC was 0.882 ± 0.115%, and it gradually decreased from the upper layer (0–20 cm) to the middle layer (20–40 cm) as 0.747 ± 0.073%, reached the peak decline as 0.692 ± 0.083% at 40–60 cm, increased to 0.755 ± 0.127% with further depth at 60–80 cm, and reached a maximum increase of 0.895 ± 0.054% at the bottom sediment layer with a depth of 80–100 cm. However, the results for the TOC of the control group (non-oyster culture area) showed that the proportion of TOC in different layers was significantly higher than that in the oyster culture area.

Similarly, the total nitrogen (TN)% values at the oyster site were 0.081 ± 0.007%, 0.075 ± 0.006%, 0.063 ± 0.004%, 0.056 ± 0.009%, and 0.073 ± 0.011% at 0–20, 20–40, 40–60, 60–80, and 80–100 cm of depth, respectively (Figure 2B). These values were significantly different from those of the reference site (Figure 2B). TN was also lower at the oyster site, and almost all five layers showed significant alleviation of TN by 29, 7, 3, 22, and 32% at sediment depths of 0–20, 20–40, 40–60, 60–80, and 80–100 cm, respectively. As shown in Figure 2C, the TOC% was alleviated by 29, 30, 22, 24, and 15% at sediment depths of 0–20, 20–40, 40–60, 60–80, and 80–100 cm, respectively. There was significant alleviation of TN at almost all five layers compared with the site without oyster farming (reference site), except for the 40- to 60-cm depth (Figure 2D).

### Stable Isotopes δ^13^C‰ and δ^15^N‰

**FIGURE 3:**
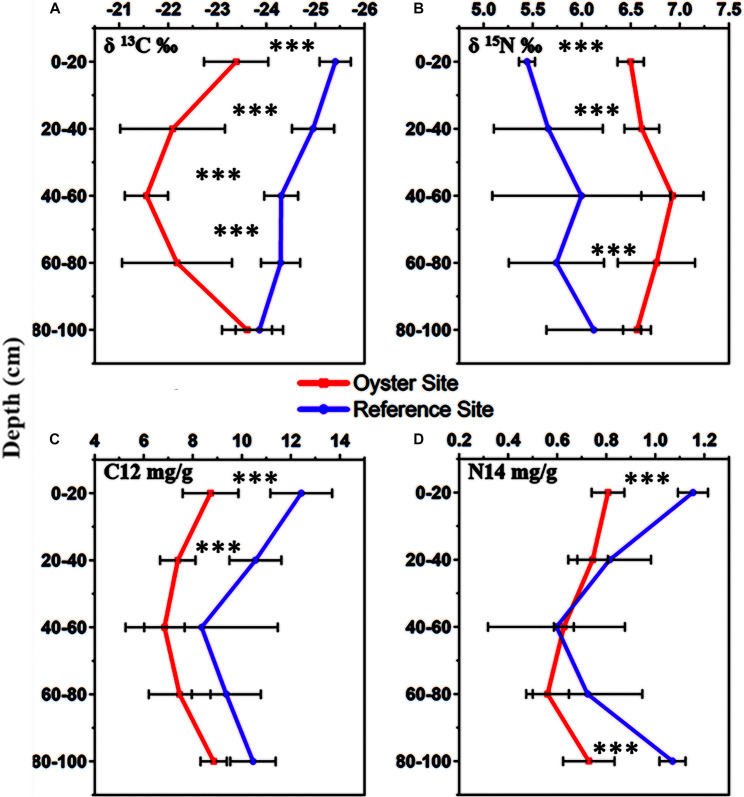
Stable isotopes of carbon δ^13^C‰ and nitrogen δ^15^N‰ in the sediments at five different levels (0–20, 20–40, 40–60, 60–80, and 80–100 cm). **(A)** Stable isotope of carbon or δ^13^C‰, **(B)** stable isotope of nitrogen or δ^15^N‰, **(C)** normal form of carbon or C12 mg/g, **(D)** normal form of nitrogen or N14 mg/g. In this image, the red color indicates the stable isotope values from the oyster site, and the purple color denotes the stable isotope values at the reference site. Error bars denote the standard deviation (SD) of the mean (*n* = 90). The statistical significance was set at *P* < 0.01 level (***).

The results from the experimental CF-IRMS analysis showed the isotopic fractionation of light and heavy carbon as well as nitrogen elements. The values of δ^13^C‰ and δ^15^N‰ ranged from −21.55 ± 0.42‰ to −23.68 ± 0.53‰ and 6.92 ± 0.30‰ to 6.51 ± 0.13‰, with an increasing concentration of heavier forms of carbon and nitrogen as compared with their lighter forms (Figures 3A,B). There was an enhanced percentage (11–12%) of δ^13^C‰ at the sub-surface, and core sediments ranging from 20 to 60 cm. However, the concentration of δ^13^C‰ decreased to 9% in the sub-bottom sediment zone, and its lowest turnover was found in the bottom sediments of the oyster-farming site (Figures 3A,B). The highest δ^15^N concentration was found in the surface sediment layers, and the lowest was in the bottom sediment layers of the oyster site (Figure 3B). The surface sediment layer (0–20 cm deep) at the oyster site contained a higher δ^13^C‰ concentration compared with the control site (*p* < 0.05).

We also measured the quantity of lighter forms of carbon and nitrogen, and there were trends similar to those for the TOC and TN results (Figures 3C,D). The stable isotope results showed a lower concentration of lighter forms, C^12^ and N^14^ mg/g, wherever the concentration of heavier forms, δ^13^C‰, and δ^15^N, was found to be higher (Figures 3C,D). There was an increase in δ^13^C‰ and δ^15^N‰ at the surface sediment layer near the suspended oyster culture site, which received direct biodeposition from hanging oysters in the water column. The concentration of δ^13^C‰ was higher where the TOC concentration was lower; however, the concentration of δ^15^N was inconsistent with δ^13^C‰ in some layers. Additionally, we noted that the concentrations of fractionated heavy carbon δ^13^C‰ and heavy nitrogen δ^15^N‰ were higher in the surface sediment, whereas the concentration of fractionated light nitrogen or N14 was lower there.

### Distribution and Correlation of Nutrients and Their Stable Isotopes

**FIGURE 4:**
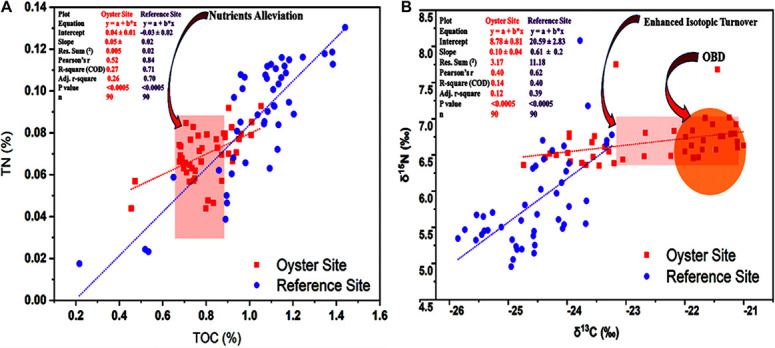
Distribution and correlation of carbon and nitrogen and their stable isotopes. **(A)** Graph shows the correlation between total organic carbon (TOC)% and TN%. Red arrow pointing toward the light red vertical rectangle indicates the alleviated nutrient values at the site under OBD influence. **(B)** Correlation between δ^13^C‰ and δ^15^N‰ is shown. The red arrow pointing toward the light red horizontal rectangle indicates the less depleted isotopic values and enhanced δ^13^C‰ and δ^15^N turnover. The red arrow pointing toward the orange circle highlights the δ^13^C‰ and δ^15^N values of OBD during nutrient alleviation. The red-colored square indicates the oyster site, and the purple-colored circle denotes the reference site. The text in the left upper corner denotes statistical comparison and contrast between the oyster and reference site.

After measurement of the statistical association between the two variables TOC% and TN%, we observed the alleviative pattern at the oyster culture site compared with the reference site (Figure 4A). The results revealed that TOC and TN exhibited a strong positive correlation at the site under OBD influence. Linear regression analysis (*r* = 0.52, *p* < 0.0005) showed the similarity between TOC and TN in scatter plots regarding the distribution trend. Alleviated values of nutrients such as TOC and TN can be seen in Figure 4A, where “a” represents the y-intercept, “b” denotes the slope, “x” denotes the independent or predictor variable, and “y” denotes the dependent or response variable. The plot equation (y = a + b^∗^ ×) shows 0.04 ± 0.01 intercept for the oyster site and –0.03 ± 0.02 for the reference site (Figure 4A). Therefore, the results regarding the distribution pattern of TOC% and TN% showed that nutrient alleviation occurred, and the correlational analysis indicated that there was a positive correlation (*r* = 0.52, *p* < 0.0005) between TOC% and TN% at the site under OBD influence.

As far as the results of distribution and correlation of stable isotopes of carbon and nitrogen were concerned, it appeared that the Pearson’s coefficient correlation between δ^13^C‰ and δ^15^N‰ was stronger at the reference site compared with the oyster site (*r* = 0.62, *p* < 0.0005). A potentially moderate correlation (*r* = 0.49, *p* < 0.0005) was also seen between δ^13^C‰ and δ^15^N‰ at the oyster farming site (Figure 4B). The results from the measurement of the statistical association between the two variables δ^13^C‰ and δ^15^N‰ showed that enhanced isotopic turnover with less depleted values existed at the oyster culture site compared with the reference site (Figure 4B). The plot equation (y = a + b^∗^ ×) showed an 8.78 ± 0.81 intercept for the oyster site and 20.59 ± 2.83 for the reference site (Figure 4B), which demonstrated enhanced isotopic turnover with less depleted values. Correlational analysis expressed moderate correlation (*r* = 0.49, *p* < 0.0005) between δ^13^C‰ and δ^15^N‰ at the site under OBD influence.

### The Abundance of Functional Genes Related to the Carbon and Nitrogen Cycle

**FIGURE 5:**
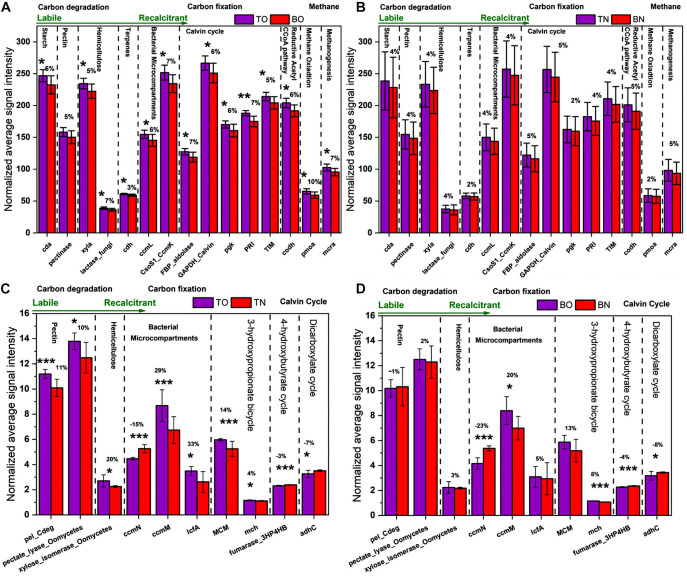
The normalized average signal intensity of functional genes related to the carbon cycle. **(A)** The figure shows significantly upregulated genes at the surface sediment portion [top layer of the oyster culturing area (TO)] compared with the bottom portion [bottom layer of the oyster culturing area (BO)] of the oyster site. **(B)** The figure shows a comparison between the surface sediment portion [top layer of the non-culturing area (TN)] compared with the bottom portion [bottom layer of the non-culturing area (BN)] of the reference site. **(C)** The figure shows significantly upregulated genes at the surface sediment portion [top layer of the oyster culturing area (TO)] of the oyster site compared with the surface portion [top layer of the non-culturing area (TN)] of the reference site. **(D)** The figure shows significantly upregulated genes at the bottom sediment portion [bottom layer of the oyster culturing area (BO)] of the oyster site compared with the bottom portion [bottom layer of the non-culturing area (BN)] of the reference site. Error bars denote the standard deviation (SD) of the mean (*n* = 16), and a % sign indicates the percent change. An asterisk indicates significant differences between groups at *p* < 0.05.

**FIGURE 6:**
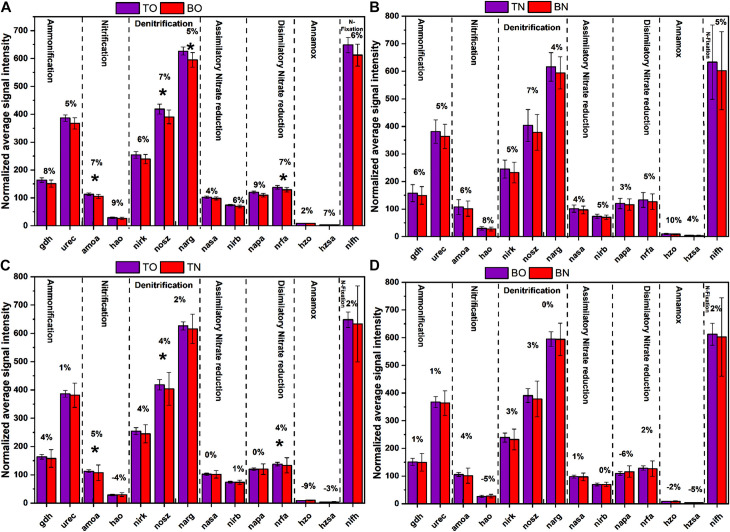
The normalized average signal intensity of functional genes related to the nitrogen cycle. **(A)** The figure shows significantly upregulated genes at the surface sediment portion (TO) compared with the bottom portion (BO) of the oyster site. **(B)** The figure shows a comparison between the surface sediment portion (TN) compared with the bottom portion (BN) of the reference site. **(C)** The figure shows significantly upregulated genes at the surface sediment portion (TO) of the oyster site compared with a surface portion (TN) of the reference site. **(D)** The figure shows significantly upregulated genes at the bottom sediment portion (BO) of the oyster site compared with the bottom portion (BN) of the reference site. Error bars denote the standard deviation (SD) of the mean (*n* = 16), and a % sign indicates the percent change. An asterisk indicates significant differences between groups at *p* < 0.05.

Microbial functional gene categories for major biogeochemical and metabolic processes were examined to understand the effects of oyster biodeposits on sediment microorganisms. One-way ANOVA and paired *t*-test analysis showed that the number of genes detected at the oyster site was significantly higher than that at the reference site (Figures 5, 6 and Supplementary Table 1). No significant difference was observed between the surface and bottom sediment layers of the reference site (Figures 5, 6). Among carbon metabolism genes, functional genes related to carbon degradation were the most abundant in all samples, followed by carbon fixation and methanogenesis genes (Figure 5). A total of 130 genes related to the carbon cycle, including 38% for carbon degradation, 45% for carbon fixation, and 17% associated with methanogenesis, were found at the site under OBD influence. The gene lactase fungi coding for hemicellulose lactose showed the highest percentage increase among labile carbon degradation genes at the oyster site. The enzyme *cda* coding for alpha amylase to degrade starch showed the second-highest percent increase with a significant difference (*p* < 0.005) at the oyster site.

**FIGURE 7:**
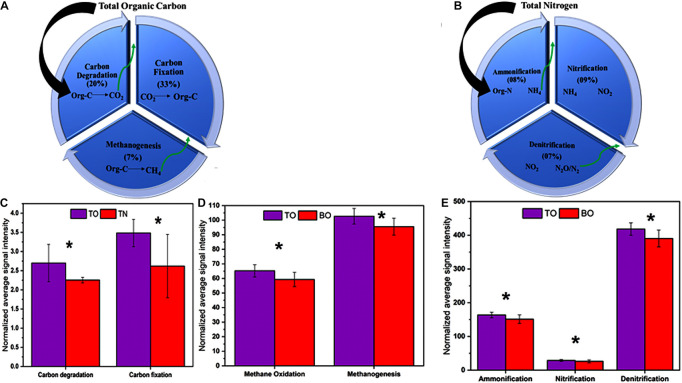
Effect on major biological processes related to carbon and nitrogen. **(A)** Percent of total organic carbon converted to CO_2_ *via* carbon degradation, reconversion of organic carbon from CO_2,_ and final degradation to methane. **(B)** Percent of total nitrogen converted to NH_4_ *via* ammonification, conversion of NH_4_ to nitrite, and final conversion of nitrite to N_2_/N_2_O gases. **(C)** Key genes involved in carbon degradation and fixation processes. **(D)** Key genes involved in methane oxidation and methanogenesis. **(E)** Key genes involved in ammonification, denitrification, and nitrification. Error bars denote the standard deviation (SD) of the mean (*n* = 16). An asterisk indicates significant differences between groups at *p* < 0.05.

The paired *t*-test analysis showed that the gene *pectate_lyase_Oomycetes* that codes for labile pectin degradation emitted significantly higher signal intensity at the oyster site. The surface oyster site receiving direct biodeposition significantly degraded both labile and recalcitrant carbon (*p* < 0.05), but the genes for labile organic matter degradation were more abundant than those for recalcitrant; therefore, labile carbon degradation was occurring at a higher rate (Figure 5). Among the abundance of carbon fixation genes, the gene *GAPDH_Calvin* coding for the enzyme glyceraldehyde-3-phosphate dehydrogenase showed significantly greater (*p* < 0.05) signal intensity at the oyster site. However, an abundance of genes coding the enzymes for methane synthesis and oxidation was noted (Figure 7). In N metabolism (Figure 6), key functional genes for ammonification, anammox, denitrification, assimilatory N reduction, dissimilatory N reduction, nitrification, and nitrification fixation were detected in all groups [the top layer of the oyster-culturing area (TO); top layer of the non-culturing area (TN); bottom layer of the oyster-culturing area (BO); and bottom layer of the non-culturing area (BN)]. The highest signal intensities were from the genes *narg* and *nifh* involved in denitrification and nitrogen fixation, indicating simultaneous nitrogen loss, removal, and sequestration. There was a significant difference among the surface oyster area and the reference site and surface vs. the bottom oyster site for the gene *amoa* involved in nitrification (*p* < 0.005). For denitrification, only *nirk*, *nosz*, and *narg* exhibited a significant difference between the oyster and reference sites. For denitrification at the reference site, there were no significant differences in the abundances of the genes *nirk*, *nosz*, and *narg.*

## Discussion

### Alleviative Effect on Total Carbon and Nitrogen at the Site Under Oyster Biodeposition Influence

Previously, it was deemed that sedimentary OM could act as an internal source of nutrients and energy, which might have important implications for the ecological sustainability of coastal marine ecosystems and eutrophication. In this work, we observed a significantly lower quantity of total carbon and nitrogen at the oyster site compared with the reference site (Figure 2). A possible reason for this was oyster biodeposits that were intermixed with sediment, which affected their textural and compositional characteristics. In past research, scientists have demonstrated that even though oyster farming may contribute to higher inputs of unfiltered and excreted organic matter at its located site, it may not always result in nutrient enrichment and eutrophication (Kellogg et al., 2013).

The Chesapeake Bay Program Scientific and Technical Advisory Committee^2^ in the year 2013 also presented a report describing the role of OBD in alleviating nutrient status. They mentioned that the nutrients supplied through oyster biodeposits might be partially released into the atmosphere and buried in deep sediments. Thus, we found lower values of TOC% and TN% due to sediment–biodeposit mix-up that constituted a different textural characteristic of surface sediment by incorporating more labile or degradable organic matter. The linear regression analysis with Pearson’s coefficient correlation showed that the distribution pattern of nutrients was greatly affected by the OBD at the oyster site. A strong positive correlation (*r* = 0.52, *p* < 0.0005) between total organic carbon and nitrogen with an intercept for oysters and for the reference site suggested their use of a common source at the places under OBD influence. The combination of OBD with sediment gave rise to the expedition and boosted the microbial communities to efficiently and actively participate in releasing nutrients from sediments back into the water column and the environment.

Previous studies suggested that the biochemical composition of oysters and other shellfish biodeposits could boost the rate of metabolic activities that convert excessively available sediment carbon and nitrogen into releasable and sequestered forms (Hoellein et al., 2015). Most filter feeders have the ability to influence deposition, transport, and the composition of suspended sediments in estuaries (Burge et al., 2016). The results from numerous studies (Hoellein and Zarnoch, 2014; Hoellein et al., 2015; Reis et al., 2017; Yang et al., 2017; Ledford et al., 2020) are in agreement with our results, i.e., OBD had an alleviative effect on sediment TOC and TN at the oyster culture site of Shenzhen Bay. Hence, it might be helpful in eutrophic management.

### Effect on Stable Isotopes δ^13^C‰ and δ^15^N‰ at the Site Under Oyster Biodeposition Influence

The surface sediment layer of the site under OBD influence showed less depleted values of δ^13^C than the reference site, suggesting that the sediment layer has been receiving a mixture of inputs from estuarine and OBD sources. However, the δ^15^N values from the surface sediment layer of the oyster site indicated that there was also an anthropogenic influence at the oyster farming area. There was an enhanced percentage of δ^13^C‰ (11–12%) at sub-surface and core sediments, which decreased to (9%) in the sub-bottom, and its lowest turnover was found in the bottom sediments of the oyster-farming site, indicating the additional presence of organic carbon.

The sub-surface sediment portion expressed less depletion and increased δ^13^C values, suggesting that the mixture of inputs from estuarine and OBD sources not only existed at the surface layer, but it also settled with increasing sediment depths. The surface sediment layer that received direct biodeposition from hanging oysters also showed increased δ^15^N‰. The change in the isotopic signature of δ^13^C‰ and δ^15^N‰, and the lower values of TOC% and TN% at the oyster culture site compared with the reference site appearing in regression analysis indicated the utilization of lighter forms of carbon and nitrogen under OBD influence.

Higher δ^13^C‰ and δ^15^N‰ in surface sediment, but lower N^14^ implied that the fractionation of carbon and nitrogen resulted in their fluctuating values. The lowest turnover of δ^13^C in the bottom sediments at the oyster-farming site indicated a greater presence of organic carbon. The possible reason behind the higher δ^13^C‰ concentration at the oyster site compared with the control site (*p* < 0.05) might be the OBD influence, which drove the surface microbial communities to expedite the metabolic process of organic carbon reduction and release.

**FIGURE 8:**
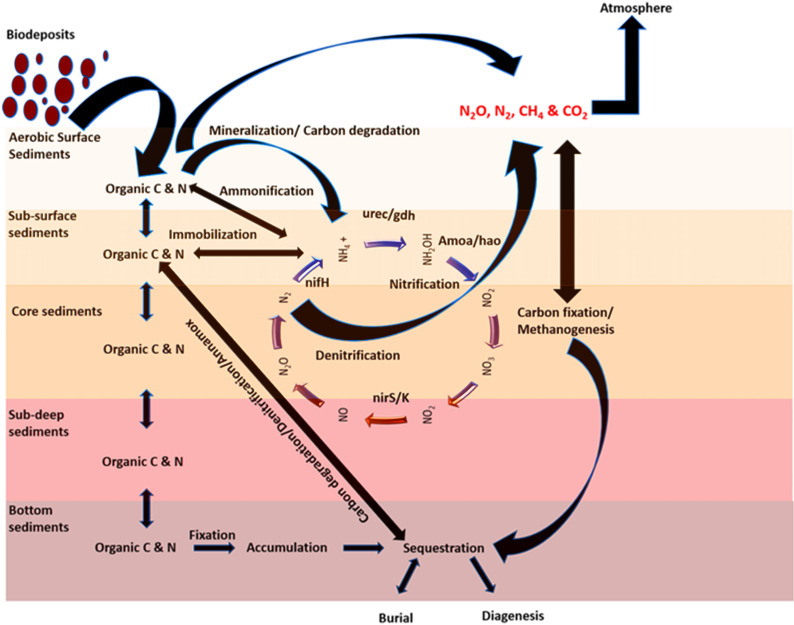
A conceptual model showing the biological process during the carbon and nitrogen cycle under an OBD process.

Additional evidence was the dynamic change in isotopic fractionation of carbon and nitrogen in the deep sediment layers of the site that was under OBD influence. We found a substantial decrease in the concentration of heavier forms of carbon and nitrogen in deep sediments (Figure 3) that indicated a greater presence of normal and lighter forms of carbon and nitrogen, which might enter into the process of burial and sequestration (Figure 8). Our reported δ^13^C and δ^15^N values are consistent with those reported in other research studies (Yang et al., 2005; Xia et al., 2014), especially a 2010 (Sampaio et al., 2010) work that mentioned the reported values of sewage effluents (δ^13^C = −22.4‰ to −26.5‰ and δ^15^N = 1.8‰ to 3.8‰) and marine organic matter (δ^13^C = −18‰ to −24‰ and δ^15^N = 4‰ to 9‰). In addition, a study on the Pearl River estuary (which is near Shenzhen Bay) also reported similar results that could support our current data (He et al., 2010). The present study has proven the fact that the use of two isotopes to characterize particulate organic matter allowed for an increased understanding of nutrient variation, containment of sources, and transformation pathways.

### The Carbon and Nitrogen Metabolic Activities of Sedimental Microbes Under Oyster Biodeposition Influence

To clarify the mechanism of OBD alleviating eutrophication, the GeoChip analysis was carried out. It was helpful to obtain further evidence to ascertain the reason behind the observed variability in sediment nutrients, particularly TOC and TN. The C cycle gene distribution in the sediment environment was significantly affected by the OBD behavior. Most of the enriched genes were associated with organic matter degradation, indicating the inter-conversion of TOC and carbon dioxide.

Upregulated genes for carbon degradation at the site under OBD influence were found in the coding of important pathway components such as *cda* (alpha amylase), *pectinase* (pectin), *xyla* (xylose isomerase), *lactase_fungi* (lactase), *cdh* (cellobiose dehydrogenase), *ccml* (carboxysome structural protein), and *csoS1_Ccmk* (α- and β-carboxysomal proteins). The gene *cda* coding for the protein enzyme alpha amylase catalyzes the degradation of starch carbohydrates; thus, the labile carbon from starch faces microbial degradation in sediments. Pectin and hemicellulose were also in the category of semi-labile and, therefore, were degraded by microbes. However, *cdh*, which codes for cellobiose dehydrogenase, acted on terpenes to bring about the microbial degradation of recalcitrant carbon. Therefore, the surface site of oysters (TO) that received direct biodeposition from oyster rafts was involved in the significant degradation of both labile and recalcitrant carbon (*p* < 0.05), but the genes for labile organic matter degradation were more abundant than those for the recalcitrant. This implies that labile carbon degradation was occurring at a higher rate (Figure 5 and Supplementary Table 1).

Among the carbon fixation pathways, characterization based on TOC degradation strategies suggested that the OBD area had a larger fraction of known r-strategists, whereas the reference area had a higher proportion of k-strategists. R-strategists preferentially consume substances with labile C, have high nutritional requirements, and exhibit rapid growth when resources are abundant. In contrast, K-strategists have slower growth rates and likely outcompete R-strategists under conditions of limited nutrient availability due to their high substrate affinity (Fierer et al., 2007).

During the carbon fixation process, at the site under OBD influence, some functional genes were significantly changed, such as *ccmM* (CO_2_-concentrating mechanisms, 55–70 kDa), *IcfA* (carbonic anhydrase), *MCM* (mm-CoA mutase), and *mch* (mesaconyl-C1-CoA hydratase). These changes indicated that the C cycle was more active at the oyster site than the reference site. The gene *MCM* was found in the subcategory of the 3-hydroxypropionate bicycle, and the gene *mch* is related to the 3-hydroxypropionate bicycle. The cyanobacterial *ccmM* gene has recently been proven as a source of increasing photosynthetic CO_2_ fixation in crop species (He et al., 2012). The enrichment of these genes at the oyster site indicated the mechanism by which inorganic carbon is converted to organic compounds through living organisms.

The possible consequences after the upregulation of the genes, as mentioned earlier, might be the accumulation, storage, sequestration, burial, and diagenesis of some portion of the sediment organic carbon. Unlike the genes mentioned above, three genes, *ccmN* (CO_2_-concentrating mechanisms, 26 kDa), *fumarase_3HP4HB* (fumarase), and *adhc* (alcohol dehydrogenase C), showed downregulation at the site under OBD influence (Figure 5C). Possible consequences might be CO_2_ fixation suppression of some portion of sediment organic carbon that needs to be degraded and released. However, we noted a significant increase in Calvin cycle genes in the OBD-influenced area (TO) compared with the OBD-less effective area (BO), suggesting more active carbon fixation in the upper sediment layers. From an ecological standpoint, changes in C utilization and fixation by the microbial community may have long-term effects on C storage and sequestration (Zhou et al., 2016). The carbon and nitrogen stable isotope values provided evidence of higher consumption and rapid turnover of normal or lighter carbon and nitrogen forms by the resident microbial communities. By gathering the results from total carbon, stable isotopes, and functional genes, we speculated that, although the area under OBD influence received more inputs of organic matter, the sediment microorganisms properly utilized it.

As far as the abundance of genes related to the nitrogen cycle was concerned, the GeoChip array showed that multiple genes were involved in nitrogen cycling (Figure 7). The majority of the genes coded the enzymes for the denitrification process, which indicated that the conversion of organic nitrogen to ammonia occurred. Assimilatory nitrate reduction (ANR) and dissimilatory nitrate reduction (DNR) might have been enhanced at the oyster area to compensate for the deficiency in organic nitrogen. In addition, ammonia monooxygenases (*amoA/B/C*) were enriched, and these are involved in ammonia oxidation and catalyze ammonia oxidation to nitrite. Ammonium production from N_2_ and nitrate may have been enhanced in the microbial community through enrichment of *nirk* (nitrite reductase-copper-containing), *nirb* (nitrite reductases-b), *nifh* (nitrogenase reductase), *napa* (nitrate reductases-a), and *nrfa* (nitrite reductase-a), thereby increasing the ammonium conversion efficiency.

Other nitrogen metabolic pathways are also affected by OBD, including the upregulated *gdh* (glutamate dehydrogenase), *urec* (urease), *hao* (hydroxylamine oxidoreductase), *nosz* (nitrous-oxide reductase), *narg* (nitrate reductase), *nasa* (nitrate-assimilating genes), *hzo* (hydrazine oxidoreductase), and *hzsa* (hydrazine synthase) (Figure 6). The nitrogen present in the organic matter can be converted to ammonia with the assistance of the *urec* and *gdh* genes. We also observed that the diminished gene pool of nitrogen metabolism genes at the reference site indicates that flourishing bacteria increased the utilization of the organic nitrogen present in the sediment. Our study assessed the sediment nutrient status in the area under OBD influence and observed an alleviated trend of nutrient overload under oyster farming from the microbial ecological perspective.

Some previous studies reported sediment nutrient alleviation from an aquatic ecosystem due to sediment denitrification (Philippot, 2002; Newell, 2004; Kellogg et al., 2013; Hoellein and Zarnoch, 2014; Hoellein et al., 2015). These studies have greatly supported our current research work (Dalsgaard et al., 2003; Giblin et al., 2013). On the whole, it was concluded that the sediments were greatly influenced by OBD input, which resulted in a more obvious alleviative effect in return. The significant enrichment of sediment nitrogen cycle genes due to increased microbial metabolic activity led to alleviated nutrients under OBD influence (Figures 5, 6, 8).

## Conclusion

The current study addressed the issue of mitigating nutrient load by conducting fieldwork *in situ* to assess the sediment nutrient status of an area under OBD influence. Oyster farming *via* its biodeposition ameliorated the distribution of total organic carbon, total nitrogen, and their stable isotopes (δ^13^C‰ and δ^15^N‰). The stable isotopes showed that different utilization forms (lighter or heavy) of carbon and nitrogen were observed in the test area and control area. The GeoChip data confirmed that the potential mechanisms of OBD on C and N cycling might have originated from increased carbon degradation ability and increased denitrification capacity of the sediments. Our results provided related evidence that the sediments were greatly influenced by OBD input, which resulted in a more obvious alleviative effect in return. The significant enrichment of sediment carbon and nitrogen cycle genes due to increased microbial metabolic activity leads to nutrient alleviation under OBD influence. Also, the OBD changed the carbon–nitrogen flux and reduced the nutrient levels, which might be beneficial for a healthy aquatic ecosystem. The molecular mechanisms of oysters as ecological engineers to mitigate nutrient load in an aquatic ecosystem deserve further study in the future.

## Data Availability Statement

The original contributions presented in the study are included in the article/Supplementary Material, further inquiries can be directed to the corresponding author/s.

## Author Contributions

AM was responsible for the conceptualization, investigation, preparation, and writing of the original draft. QY organized the teamwork in the field area and analyzed the environmental factors. XS and JS were in charge of the data analysis and software application. XL collected the samples and participated in the field experiments. JZ and Z-HC read and revised the manuscript and were in charge of the funding acquisition and project administration. All authors contributed to the article and approved the submitted version.

## Conflict of Interest

The authors declare that the research was conducted in the absence of any commercial or financial relationships that could be construed as a potential conflict of interest.

## Publisher’s Note

All claims expressed in this article are solely those of the authors and do not necessarily represent those of their affiliated organizations, or those of the publisher, the editors and the reviewers. Any product that may be evaluated in this article, or claim that may be made by its manufacturer, is not guaranteed or endorsed by the publisher.

## Footnotes

1
http://ieg.ou.edu/microarray/

2
https://www.chesapeake.org/stac/

## References

[B1] Bowen J. L. Valiela I. (2001). Historical changes in atmospheric nitrogen deposition to Cape Cod, Massachusetts, USA. *Atmospheric Environ.* 35 1039–1051. 10.1016/s1352-2310(00)00331-9

[B2] Burge C. Closek C. Friedman C. Groner M. Jenkins C. Shore A. (2016). The use of filter-feeders to manage disease in a changing world. *Integr. Comparat. Biol.* 56:icw048. 10.1093/icb/icw048 27371383

[B3] Chan Y. Van Nostrand J. D. Zhou J. Pointing S. B. Farrell R. L. (2013). Functional ecology of an antarctic dry valley. *Proc. Natl. Acad. Sci. U.S.A.* 110 8990–8995. 23671121 10.1073/pnas.1300643110 PMC3670347

[B4] Cornwell J. C. Kemp W M. Kana T. M. (1999). Denitrification in coastal ecosystems: methods, environmental controls, and ecosystem level controls, a review. *Aquatic Ecol.* 33 41–54.

[B5] Cunha M. E. Quental-Ferreira H. Parejo A. Gamito S. Ribeiro L. Moreira M. (2019). Methodology for assessing the individual role of fish, oyster, phytoplankton and macroalgae in the ecology of integrated production in earthen ponds. *MethodsX* 6 2570–2576. 10.1016/j.mex.2019.10.016 31763188 PMC6861580

[B6] Dalsgaard T. Canfield D. E. Petersen J. Thamdrup B. Acuña-González J. (2003). N2 production by the anammox reaction in the anoxic water column of golfo dulce. Costa Rica. *Nature* 422 606–608. 10.1038/nature01526 12686998

[B7] Diaz R. J. Rosenberg R. (1995). Oceanography and marine biology: an annual review of marine benthic hypoxia: a review of its ecological effects and the behavioral responses of benthic macrofauna. *London U.K. Univ. College London Press* 33 245–303.

[B8] Diaz R. J. Rosenberg R. (2008). Spreading dead zones and consequences for marine ecosystems. *Science* 321 926–929. 10.1126/science.1156401 18703733

[B9] Fierer N. Bradford M. A. Jackson R. B. (2007). Toward an ecological classification of soil bacteria. *Ecology* 88 1354–1364. 10.1890/05-1839 17601128

[B10] Forrest B. M. Keeley N. B. Hopkins G. A. Webb S. C. Clement D. M. (2009). Bivalve aquaculture in estuaries: review and synthesis of oyster cultivation effects. *Aquaculture* 298 1–15. 10.1016/j.aquaculture.2009.09.032

[B11] Gao X. Yang Y. Wang C. (2012). Geochemistry of organic carbon and nitrogen in surface sediments of coastal bohai bay inferred from their ratios and stable isotopic signatures. *Mar. Pollut. Bull.* 64 1148–1155. 10.1016/j.marpolbul.2012.03.028 22537970

[B12] Gautreau E. Volatier L. Nogaro G. Gouze E. Mermillod-Blondin F. (2020). The influence of bioturbation and water column oxygenation on nutrient recycling in reservoir sediments. *Hydrobiologia* 847 1027–1040. 10.1007/s10750-019-04166-0

[B13] Giblin A. E. Tobias C. R. Song B. Weston N. Banta G. T. Rivera V. (2013). The importance of dissimilatory nitrate reduction to ammonium (DNRA) in the nitrogen cycle of coastal ecosystems. *Oceanography* 26 124–131. 10.5670/oceanog.2013.54

[B14] Giles H. Pilditch C. A. (2006). Effects of mussel *Perna canaliculus* biodeposit decomposition on benthic respiration and nutrient fluxes. *Mar. Biol.* 150 261–271. 10.1007/s00227-006-0348-7

[B15] Glibert P. Seitzinger S. Heil C. Burkholder J. Parrow M. Codispoti L. (2005). The role of eutrophication in the global proliferation of harmful algal blooms. *Oceanography* 18 198–209. 10.5670/oceanog.2005.54

[B16] Grabowski J. Peterson C. (2007). Restoring oyster reefs to recover ecosystem services. *Theor. Ecol. Ser.* 4:e80017–7. 10.1016/S1875-306X(07)80017-7

[B17] Grasshoff K. Ehrhardt M. Kremling K. (1985). Methods of seawater analysis. second, revised and extended edition. *Int. Revue Der Gesamten Hydrobiol. Hydrogr.* 70 302–303. 10.1002/iroh.19850700232

[B18] He B. Dai M. Huang W. Liu Q. Chen H. Xu L. (2010). Sources and accumulation of organic carbon in the pearl river estuary surface sediment as indicated by elemental, stable carbon isotopic, and carbohydrate compositions. *Biogeosciences* 7:3343. 10.5194/bg-7-3343-2010

[B19] He Z. Van Nostrand J. D. Zhou J. (2012). Applications of functional gene microarrays for profiling microbial communities. *Curr. Opin. Biotechnol.* 23 460–466. 10.1016/j.copbio.2011.12.021 22226464

[B20] Hoellein T. J. Zarnoch C. B. (2014). Effect of eastern oysters *Crassostrea virginica* on sediment carbon and nitrogen dynamics in an urban estuary. *Ecol. Appl.* 24 271–286. 10.1890/12-1798.1 24689140

[B21] Hoellein T. J. Zarnoch C. B. Grizzle R. E. (2015). Eastern oyster *Crassostrea virginica* filtration, biodeposition, and sediment nitrogen cycling at two oyster reefs with contrasting water quality in great bay estuary. *New Hampshire U.S.A. Biogeochem.* 122 113–129. 10.1007/s10533-014-0034-7

[B22] Hurt R. A. Qiu X. Wu L. Roh Y. Palumbo A. V. Tiedje J. M. (2001). Simultaneous recovery of RNA and DNA from soils and sediments. *Appl. Environ. Microbiol.* 67:4495. 10.1128/aem.67.10.4495-4503.2001 11571148 PMC93195

[B23] Jennifer H. Just C. ÂN. Ivan V. (2003). Eelgrass *Zostera marina* loss in temperate estuaries: relationship to land-derived nitrogen loads and effect of light limitation imposed by algae. *Mar. Ecol. Prog. Ser.* 247 59–73. 10.3354/meps247059

[B24] Ke Z. Tan Y. Huang L. Zhao C. Jiang X. (2017). Spatial distributions of δ13C & δ15N and C/N ratios in suspended particulate organic matter of a bay under serious anthropogenic influences: daya bay. China. *Mar. Pollut. Bull.* 114 183–191. 27590874 10.1016/j.marpolbul.2016.08.078

[B25] Kellogg M. Cornwell J. Owens M. Paynter K. (2013). Denitrification and nutrient assimilation on a restored oyster reef. *Mar. Ecol. Prog. Ser.* 480 1–19. 10.3354/meps10331

[B26] Kemp W. M. Boynton W. R. Adolf J. E. Boesch D. F. Boicourt W. C. Brush G. (2005). Eutrophication of chesapeake bay: historical trends and ecological interactions. *Mar. Ecol. Prog. Ser.* 303 1–29. 10.3354/meps303001

[B27] Ledford T. C. Mortazavi B. Tatariw C. Mason O. U. (2020). Elevated nutrient inputs to marshes differentially impact carbon and nitrogen cycling in two northern Gulf of Mexico saltmarsh plants. *Biogeochemistry* 149 1–16. 10.1007/s10533-020-00656-9

[B28] Loren D. C. Robert D. B. David B. Ray G. Mark W. L. Martin H. P. (2007). Ecosystem services related to oyster restoration. *Mar. Ecol. Prog. Ser.* 341 303–307. 10.3354/meps341303

[B29] Lotze H. K. Lenihan H. S. Bourque B. J. Bradbury R. H. Cooke R. G. Kay M. C. (2006). Depletion, degradation, and recovery potential of estuaries and coastal seas. *Science* 312:1806. 10.1126/science.1128035 16794081

[B30] Marzocchi U. Bonaglia S. Zaiko A. Quero G. M. Vybernaite-Lubiene I. Politi T. (2021). Zebra mussel holobionts fix and recycle nitrogen in lagoon sediments. *Front. Microbiol.* 11:610269. 33542710 10.3389/fmicb.2020.610269 PMC7851879

[B31] Newell R. I. E. (1996). “Mechanisms and physiology of larval and adult feeding,” in *The Eastern Oyster Crassostrea Virginica*, eds Kennedy V. S. Newell R. I. E. Eble A. F. (College Park, MD: Maryland Sea Grant College).

[B32] Newell R. I. E. (2004). Ecosystem influences of natural and cultivated populations of suspension-feeding bivalve molluscs: a review. *J. Shellfish Res.* 23 51–61.

[B33] Newell R. I. E. Fisher T. R. Holyoke R. R. Cornwell J. C. (2005). “Influence of eastern oysters on nitrogen and phosphorus regeneration in chesapeake bay, USA,” in *The Comparative Roles of Suspension-Feeders in Ecosystems*, eds Dame R. F. Olenin S. (Dordrecht: Springer), 93–120. 10.1007/1-4020-3030-4_6

[B34] Newell R. I. E. Koch E. W. (2004). Modeling seagrass density and distribution in response to changes in turbidity stemming from bivalve filtration and seagrass sediment stabilization. *Estuaries* 27 793–806. 10.1007/bf02912041

[B35] Nixon S. W. (1995). Coastal marine eutrophication: a definition, social causes, and future concerns. *Ophelia* 41 199–219. 10.1080/00785236.1995.10422044

[B36] Oades J. M. Kirkman M. A. Wagner G. H. (1970). The use of gas-liquid chromatography for the determination of sugars extracted from soils by sulfuric acid. *Soil Sci. Soc. Am. J.* 34 230–235. 10.2136/sssaj1970.03615995003400020017x

[B37] Paerl H. W. (1997). Coastal eutrophication and harmful algal blooms: importance of atmospheric deposition and groundwater as new nitrogen and other nutrient sources. *Limnol. Oceanogr.* 42 1154–1165. 10.4319/lo.1997.42.5_part_2.1154

[B38] Peterson T. Toews H. N. J. Robinson C. Harrison P. (2007). Nutrient and phytoplankton dynamics in the queen charlotte islands Canada during the summer upwelling seasons of 2001-2002. *J. Plankton Res.* 29 219–239. 10.1093/plankt/fbm010

[B39] Philippot L. (2002). Denitrifying genes in bacterial and archaeal genomes. *Biochim. et Biophys. Acta (BBA) Gene Struct. Exp.* 1577 355–376. 10.1016/s0167-4781(02)00420-7 12359326

[B40] Rabalais N. N. Díaz R. J. Levin L. A. Turner R. E. Gilbert D. Zhang J. (2010). Dynamics and distribution of natural and human-caused hypoxia. *Biogeosciences* 7 585–619. 10.5194/bg-7-585-2010

[B41] Reis C. R. G. Nardoto G. B. Oliveira R. S. (2017). Global overview on nitrogen dynamics in mangroves and consequences of increasing nitrogen availability for these systems. *Plant Soil* 410 1–19. 10.1007/s11104-016-3123-7

[B42] Rovira P. Vallejo V. R. (2000). Examination of thermal and acid hydrolysis procedures in characterization of soil organic matter. *Commun. Soil Sci. Plant Anal.* 31 81–100. 10.1080/00103620009370422

[B43] Sampaio L. Freitas R. Máguas C. Rodrigues A. Quintino V. (2010). Coastal sediments under the influence of multiple organic enrichment sources: an evaluation using carbon and nitrogen stable isotopes. *Mar. Pollut. Bull.* 60 272–282. 10.1016/j.marpolbul.2009.09.008 19796774

[B44] Xia B. Cui Y. Chen B. Cui Z. Qu K. Ma F. (2014). Carbon and nitrogen isotope analysis and sources of organic matter in surface sediments from the sanggou bay and its adjacent areas, China. *Acta Oceanol. Sin.* 33 48–57. 10.1007/s13131-014-0574-7

[B45] Yan Q. Bi Y. Deng Y. He Z. Wu L. Van Nostrand J. D. (2015). Impacts of the three gorges dam on microbial structure and potential function. *Sci. Rep.* 5 1–9. 10.1038/srep08605 PMC4342553 25721383

[B46] Yang H. Zhou Y. Mao Y. Li X. Liu Y. Zhang F. (2005). Growth characters and photosynthetic capacity of *Gracilaria lemaneiformis* as a biofilter in a shellfish farming area in sanggou bay, China. *J. Appl. Phycol.* 17 199–206. 10.1007/s10811-005-6419-1

[B47] Yang W. Qiao Y. Li N. Zhao H. Yang R. Leng X. (2017). Seawall construction alters soil carbon and nitrogen dynamics and soil microbial biomass in an invasive *Spartina alterniflora* salt marsh in eastern China. *Appl. Soil Ecol.* 110 1–11. 10.1016/j.apsoil.2016.11.007

[B48] Zhou X. Zhou L. Nie Y. Fu Y. Du Z. Shao J. (2016). Similar responses of soil carbon storage to drought and irrigation in terrestrial ecosystems but with contrasting mechanisms: a meta-analysis. *Agric. Ecosyst. Environ.* 228 70–81. 10.1016/j.agee.2016.04.030

