# Supplementary material for: Oyster Biodeposition Alleviates Sediment Nutrient Overload: A Case Study at Shenzhen Bay, China
Source: Front Microbiol. 2021 Nov 10;12:716201. doi: 10.3389/fmicb.2021.716201 (PMC8631438; doi:10.3389/fmicb.2021.716201)
Supplement: Supplementary file 1 [file Data_Sheet_1.docx]

**Supplementary information**


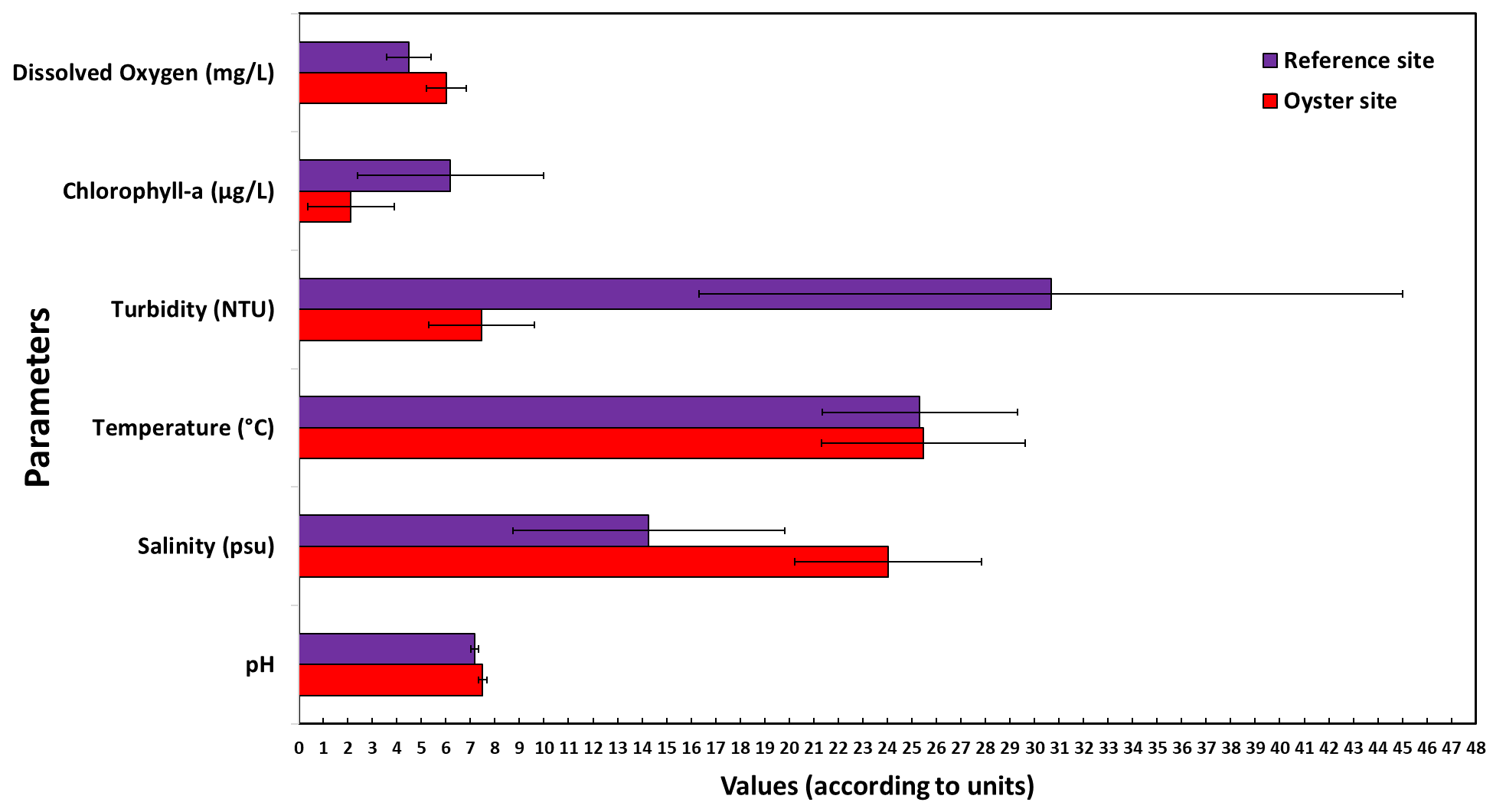


**Figure S1: This figure shows the physical conditions/parameters of Study site**

**
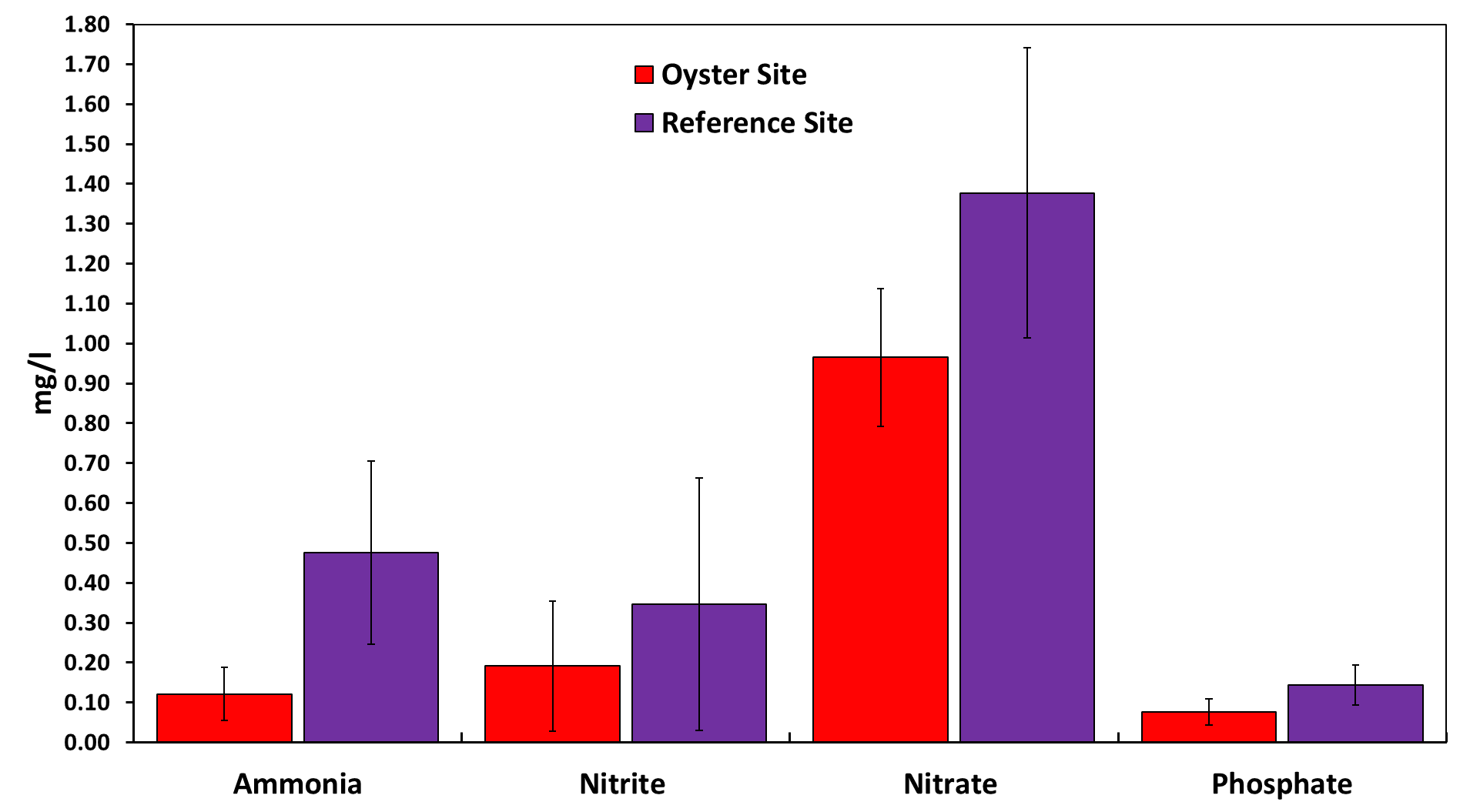
**

**Figure S2: This figure shows the inorganic nutrient level in the water column of studied sites.**

| *Groups* | *Count* | *Sum* | *Average* | *Variance* |  |  |
| --- | --- | --- | --- | --- | --- | --- |
| GAPDH_Calvin(TO) | 8 | 2134.08 | 266.76 | 144.0217 |  |  |
| CsoS1_CcmK(TN) | 8 | 2058.961 | 257.3701 | 2234.535 |  |  |
| GAPDH_Calvin(TN) | 8 | 2054.278 | 256.7847 | 1510.498 |  |  |
| CsoS1_CcmK(TO) | 8 | 2013.546 | 251.6933 | 157.6611 |  |  |
| cda(TO) | 8 | 1975.129 | 246.8912 | 103.2799 |  |  |
| cda(TN) | 8 | 1909.177 | 238.6471 | 2383.932 |  |  |
| xyla(TO) | 8 | 1876.469 | 234.5586 | 78.18671 |  |  |
| xyla(TN) | 8 | 1867.863 | 233.4829 | 1458.816 |  |  |
| TIM(TO) | 8 | 1713.297 | 214.1621 | 49.29199 |  |  |
| TIM(TN) | 8 | 1686.034 | 210.7542 | 782.0966 |  |  |
| codh(TO) | 8 | 1633.266 | 204.1582 | 62.0626 |  |  |
| codh(TN) | 8 | 1608.99 | 201.1238 | 814.8727 |  |  |
| PRI(TO) | 8 | 1503.622 | 187.9527 | 20.37309 |  |  |
| PRI(TN) | 8 | 1460.65 | 182.5812 | 575.1373 |  |  |
| pgk(TO) | 8 | 1362.119 | 170.2648 | 36.07106 |  |  |
| pgk(TN) | 8 | 1299.997 | 162.4997 | 502.2243 |  |  |
| pectinase (pectate_lyase)(TO) | 8 | 1266.532 | 158.3165 | 57.56723 |  |  |
| ccmL(TO) | 8 | 1238.346 | 154.7933 | 47.50598 |  |  |
| pectinase (pectate_lyase)(TN) | 8 | 1237.821 | 154.7276 | 600.2982 |  |  |
| ccmL(TN) | 8 | 1201.07 | 150.1338 | 505.8258 |  |  |
| FBP_aldolase(TO) | 8 | 1020.109 | 127.5137 | 28.17363 |  |  |
| FBP_aldolase(TN) | 8 | 977.6809 | 122.2101 | 404.9814 |  |  |
| mcra(TO) | 8 | 821.4628 | 102.6828 | 32.80087 |  |  |
| mcra(TN) | 8 | 785.9896 | 98.2487 | 332.9381 |  |  |
| pmoa(TO) | 8 | 521.9196 | 65.23995 | 20.24679 |  |  |
| cdh(TO) | 8 | 488.3726 | 61.04657 | 1.206089 |  |  |
| pmoa(TN) | 8 | 468.7267 | 58.59083 | 127.2033 |  |  |
| cdh(TN) | 8 | 464.5333 | 58.06666 | 21.88636 |  |  |
| lactase_fungi(TO) | 8 | 310.8286 | 38.85358 | 3.891463 |  |  |
| lactase_fungi(TN) | 8 | 299.6444 | 37.45555 | 41.46031 |  |  |
| ANOVA |  |  |  |  |  |  |
| *Source of Variation* | *SS* | *df* | *MS* | *F* | ***P-value*** | *F crit* |
| Between Groups | 1219133.265 | 29 | 42039.08 | 95.98659 | **0.000** | 1.521631 |
| Within Groups | 91973.32662 | 210 | 437.9682 |  |  |  |
| Total | 1311106.592 | 239 |  |  |  |  |

**Table S1: One way ANOVA (single factor) shows significant variances of carbon cycle genes between the Oyster site (TO) and Reference site (TN). P-value < 0.05 stands statistically significant.**
